# The impact of dyadic interventions on psycho-social outcomes for stroke patients and their caregivers: a systematic review and meta-analysis

**DOI:** 10.3389/fpubh.2025.1583621

**Published:** 2025-05-09

**Authors:** Xinyue Xing, Liping Pu, Wanyue Hu, Yu Xiao, Hongling Xiao

**Affiliations:** ^1^The School of Graduate, Tianjin University of Traditional Chinese Medicine, Tianjin, China; ^2^Suzhou Vocational Health College, Suzhou, China; ^3^The School of Nursing, Zhejiang Chinese Medical University, Hangzhou, China

**Keywords:** stroke, caregivers, dyad, systematic review, meta-analysis, randomized controlled trial

## Abstract

**Aims:**

To describe the details of dyadic interventions and summarize the current evidence on supporting dyadic interventions for psycho-social outcomes of stroke patients and their caregivers.

**Design:**

A systematic review and meta-analysis based on PRISMA guidelines.

**Data sources:**

Five English databases (PubMed, Web of Science, CINAHL, Embase and Cochrane Library) were searched to identify eligible studies published from the inception to October 15, 2024.

**Methods:**

Two reviewers independently screened the literature in accordance with the selection criteria. The risk of bias of the included studies was assessed using Cochrane RoB 2.0. Relevant information was extracted, narrative synthesis was conducted and the meta-analysis was carried out using Review Manager 5.4 soft.

**Results:**

A total of 28 literature were identified and included in this review. These interventions focused mainly on the provision of stroke related knowledge, promotion of family relationships and relief of negative emotions by a face-to-face mode. The outcome indicators can be grouped into three dimensions based on the developmental contextual coping model: dyadic appraisal, dyadic coping and dyadic adjustments. The results of meta-analysis showed that dyadic interventions significantly improved quality of life and coping capacity of patients, promoted family function of caregivers and alleviated caregiver-related burden.

**Conclusion:**

These findings highlighted the positive outcomes of dyadic interventions that focused on patients and their caregivers coping with stroke. However, the effectiveness of interventions is not absolute, the evaluation system of intervention effect needs to be improved and demand-driven interventions need to be developed urgently. Therefore, further large-scale randomized controlled trials with a high-quality design are warranted.

**Systematic review registration:**

https://www.crd.york.ac.uk/PROSPERO/, CRD42024621297.

## 1 Introduction

Stroke, a common chronic disease, ranks as the second major cause of disability and mortality globally, and stands as the primary cause of premature death in China (1). Due to accelerated population ageing and changing lifestyles, the prevalence of stroke is gradually increasing. It is anticipated that the incidence of cerebrovascular diseases in China will increase by approximately 50% by 2030 compared to 2010 (2). Over 60% of stroke survivors encounter some forms of dysfunction 3 months post-stroke, and the recurrence rate can reach as much as 41% within 5 years of onset (3). Statistically, the yearly medical expenditure for stroke in China amounts to approximately 40 billion RMB, a figure tenfold that of cardiovascular disease (4). The high morbidity, recurrence, disability, and economic burden of stroke impose a significant burden on the healthcare system and the whole of society. Consequently, there is an urgent need for a scientific and effective stroke management strategy to mitigate the current challenging situation.

With characteristics of the disease, stroke has emerged as a persistent source of stress for both patients and their caregivers. Patients often experience varying degrees of disability after a stroke. According to the American Stroke Association (AStA), approximately one-third of stroke survivors endure serious speech impairments, and half experience hand function loss (5). Additionally, physical condition can easily trigger psychological problems, such as anxiety and depression. It is reported that 21% of patients experience depression 1 year after stroke (6). A meta-analysis indicated that depressive disorder appeared in 33.5% of stroke patients (7). As the primary carers for patients undergoing home rehabilitation post-discharge, caregivers take a significant responsibility. Ongoing care tasks impact their normal employment, leisure pursuits, and social interactions, thereby subjecting them to considerable social pressures and psychological distress (8). Rigby’s study showed that the prevalence of caregiver burden was 25–54% and remained elevated for an indefinite period following stroke (9). Loh’s meta-analysis revealed that 40.2% of caregivers had obvious depressive symptoms (10). It can clearly be seen that stroke exerts profound adverse impacts on both patients and their caregivers.

To improve the poor health of stroke patients and their caregivers, scholars have introduced various interventions. Alexopoulos developed Ecosystem Focused Therapy (EFT) to reduce depressive symptoms and ameliorate disability for stroke survivors (11). Kendall implemented self-management education for stroke patients and found that the intervention improved rehabilitation outcomes in the short-term (12). Moreover, a strength-oriented psycho-education focused on family caregivers alleviated caring burden, and improved caregiving competence (13). However, the above interventions are aimed solely at patients or their caregivers.

According to Interdependence Theory, the essence of relationships lies in the interactions among social individuals and the interwoven nature of their behaviors (14). Stroke, a common disease of patients and their caregivers, fosters a dynamic where dyads become interdependent throughout the protracted journey of managing the illness. It is logical to assume that health outcomes for both stroke patients and caregivers are interlinked (15). Therefore, considering stroke patient and caregiver as a dyad, interventions aimed at the dyad may be proved more impactful than those directed solely at individuals, with the expectation that the benefits of such interventions will be maximized. At present, more studies have explored the impact of dyadic interventions on stroke patients and their caregivers. For example, Mou et al. implemented family-focused dyadic psycho-educational intervention and found that the program had positive effects on dyadic relationship, caregiver burden and coping ability (16). Lin et al. demonstrated that dyadic intervention was effective in improving quality of life (17). Of the existing reviews of dyadic interventions, Pucciarelli conducted a systematic review to explore the efficacy of dyadic educational intervention, but the review’s outcome measures were somewhat constrained (18). Mou’s (19) and Zhang’s (20) systematic reviews imposed limitations on the types of interventions included, which could result in an incomplete assessment. It can be seen that there is currently limited evidence of dyadic interventions for stroke patients and their caregivers.

In 2007, Berg (21) proposed the developmental contextual coping model of couples coping with chronic illness which emphasized that both partners perceive, evaluate and communicate pressure together in the process of stress coping, so as to maintain the stability of the relationship. The model includes three key elements: dyadic appraisal, dyadic coping and dyadic adjustment and posits that the process of managing chronic diseases starts with dyadic appraisal of the disease, and ultimately progresses to dyadic adjustment and adaptation via dyadic coping mechanisms. The duality of the model determines that we can divide the outcome indicators of dyadic interventions into three categories. Already scholars have encapsulated the outcomes of dyadic interventions for couples managing cancer based on the model (22). On balance, we believe that the model is equally applicable to stroke patients and their caregivers, who exist in an interdependent duality. Consequently, our study has summarized outcome indicators grounded in the model.

Given the dyadic characterization of the interactions between stroke patients and caregivers and the limitations of existing reviews, it is essential to systematically explore the details of dyadic interventions that have been developed and put into practice, focusing on the delivery modes, contents, duration, and outcome assessments, which will provide a foundation of evidence for the evolution and refinement of future dyadic intervention programs.

## 2 Methods

This systematic review and meta-analysis was structured in accordance with the PRISMA checklist (23), and the review protocol was registered in PROSPERO (CRD42024621297).

### 2.1 Search strategy

Five electronic databases (PubMed; Web of Science; CINAHL; Embase; Cochrane Library) were systematically searched from the inception to October 15, 2024 in order to screen the relevant studies as many as possible. Additional records were identified from the reference lists of all relevant articles and a manual search of relevant journals. Search keywords were as follows: (a) stroke, apoplexy, hemorrhagic stroke, ischemic stroke, brain infarction, cerebrovascular disorders, etc.; (b) caregiver*, family caregiver, informal caregiver, partner*, dyad*, dyadic, etc.; and (c) dyadic intervention, dyadic coping, dyadic management, cognitive behavioral therapy, psycho-social intervention, health education, etc. The details of the search strategies of databases are presented in Supplementary Table 1.

### 2.2 Selection criteria

#### 2.2.1 Inclusion criteria

Subjects (P): patients diagnosed with stroke and their informal caregivers (≥18 years). Informal caregivers are family members of patients who offer daily care and emotional support without any form of payment. Intervention (I): non-pharmacological interventions centered on the dyads of stroke patients and their family caregivers, encompassing descriptions of the intervention’s content and its impact. Control group (C): usual care. Outcomes (O): psycho-social outcomes including depression, functional ability, quality of life, quality of relationship, family function and caregiver burden, et al. Study type (S): randomized controlled trials (RCTs).

#### 2.2.2 Exclusion criteria

The intervention targets exclusively one of dyads; papers not written in English; papers that are reviews, abstracts, letters, conference proceedings, or protocols; republished or similar studies; and papers for which the full text is unavailable.

### 2.3 Study selection

Two mutually blinded researchers screened titles and abstracts of all the retrieved literature according to the inclusion and exclusion criteria, then the full texts of potentially relevant literature were independently reviewed. If there were any uncertain situations, a third researcher was asked to make judgements.

### 2.4 Quality appraisal

Two independent researchers assessed the quality of the included studies. In cases of disagreement, they conferred with a third researcher to achieve a consensus. The Cochrane risk of bias tool, encompassing six domains (selection bias, performance bias, detection bias, attrition bias, reporting bias, and other biases), was employed for RCTs, with each domain rated as low, high, or unclear risk of bias (24).

### 2.5 Data extraction and analysis

Data of each included literature were extracted by researchers with a structured data extraction form that included information about author, country, published time, study type, participants and sample size, intervention program and implementer, intervention duration, conceptual framework, measurement time points and outcomes. The extracted data were cross-checked and finalized with those obtained by another researcher.

Outcome measures in the review were divided into two aspects, individual outcomes (patients’ and caregivers’) and dyadic outcomes. For stroke patients, outcomes consisted mainly of functional independence, depression, self-efficacy and quality of life. For caregivers, outcomes were caregiver-related burden, depression, self-efficacy and quality of life. Dyadic outcomes included relationship quality, family function and dyadic coping. Meanwhile, outcome indicators were categorized based on three elements of the developmental contextual coping model.

Meta-analysis was conducted using RevMan 5.4. Due to differences in the measurements tools for the same indicators, the content of interventions and measurement times, we used standard mean difference (SMD) and 95% confidence interval (CI) to synthesize the pooled effects. The statistical heterogeneity was judged using the chi-square test and I^2^. If I^2^ > 50%, it indicates that there is obvious heterogeneity. For obvious heterogeneity, we used subgroup analysis and sensitivity analysis to identify the source of heterogeneity.

## 3 Results

### 3.1 Search results

**Figure 1:**
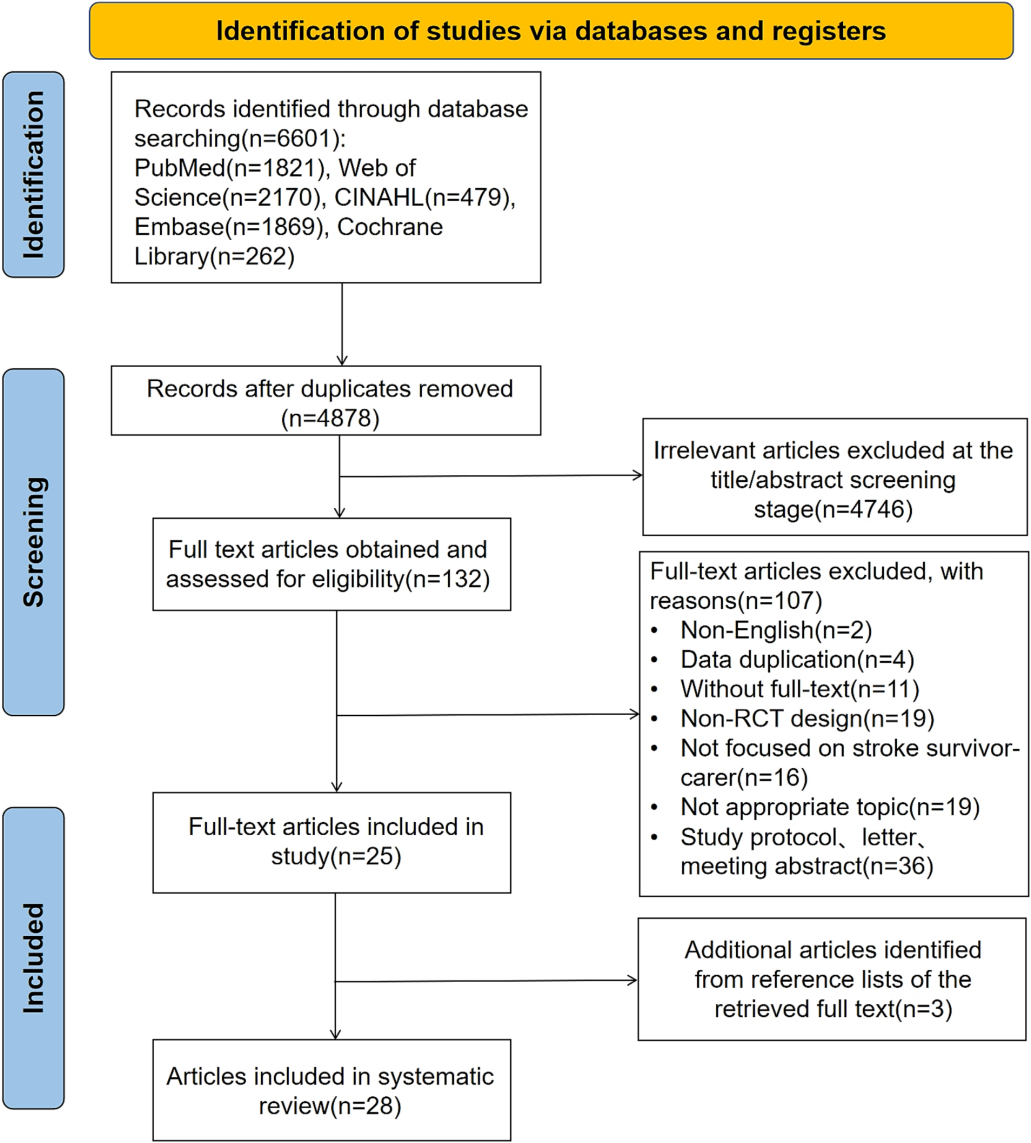
PRISMA flow diagram of included studies.

The process of study search and selection is summarized in Figure 1. A total of 6,601 literature were identified from the databases; after the duplicates (*n* = 1723) were removed using EndNote X9, 4,878 were retained for checking their relevance to the review. After titles and abstracts were screened, 4,746 irrelevant literature were excluded. The full texts of 132 literature were retrieved for screening with the selection criteria. The review included 25 eligible literature (16, 17, 25–47), supplemented by an additional 3 literature (48–50) manually retrieved, yielding a total of 28 literature for analysis.

### 3.2 Risk of bias assessment

**Figure 2:**
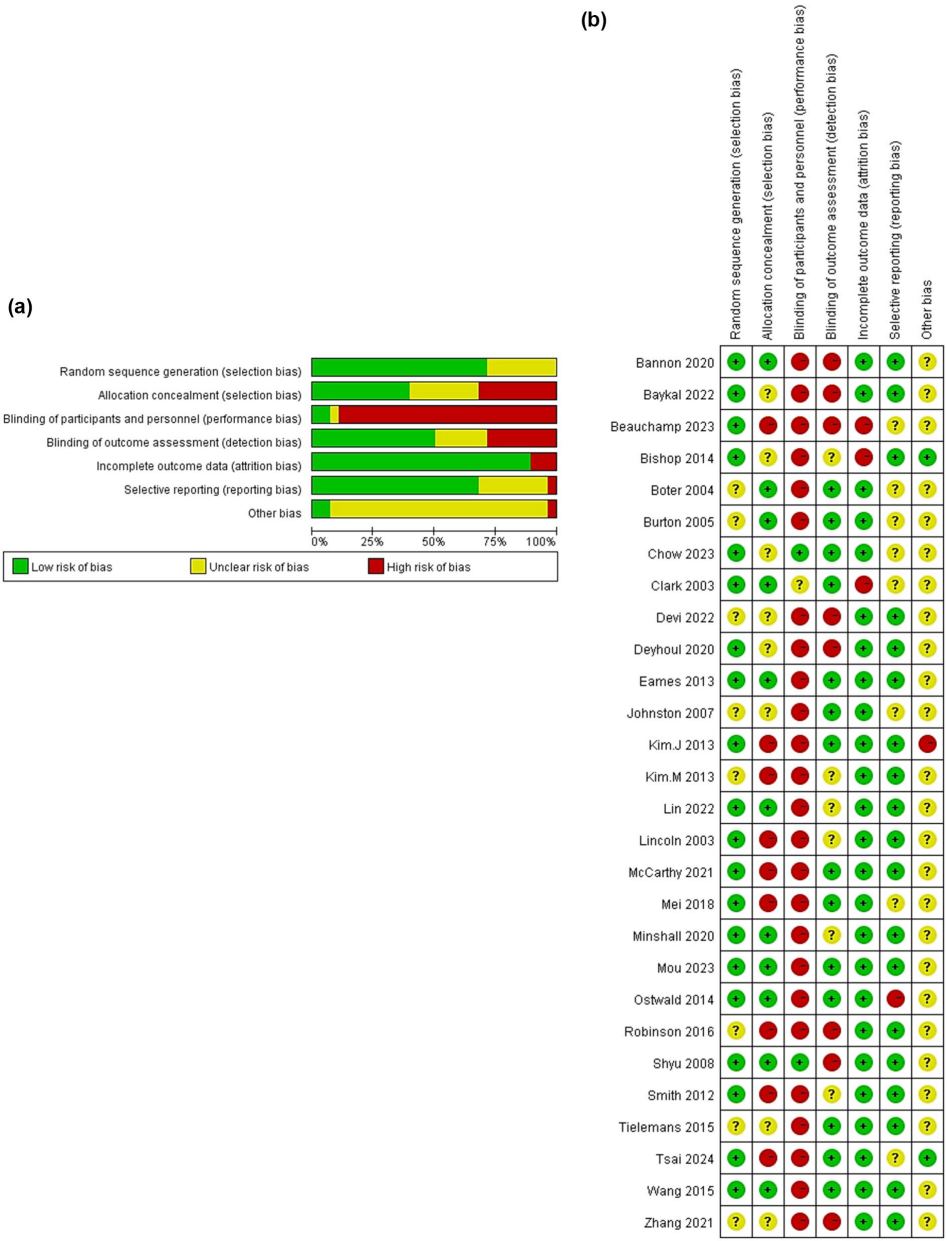
Risk of bias. Overall **(a)** and assessment of individual studies **(b)**.

Figure 2a summarizes the overall risk of bias of the 28 included studies, and Figure 2b reports the assessment results of six domains for individual studies. Four studies (35, 39, 45, 48) did not explain the method of random sequence generation in detail, four studies (26, 29, 38, 50) did not specify how to implement allocation hiding, and four studies (28, 30, 44, 49) did not describe these two parts in detail, which indicated the possibility of selection bias. The subjects or investigators in nine studies (25, 27, 31, 34, 35, 39, 40, 42, 46) may have been able to predict the allocation outcomes, suggesting a high risk of bias. 25 studies did not blind the study subject and/or the intervention provider and had a high risk of implementation bias. One study (47) did not mention whether the study subjects or intervention providers were blinded, indicating possible implementation bias. Eight studies (27–30, 32, 35, 43, 50) did not use assessments of outcome measures that were done by blinded raters, which implied a risk of measurement bias. Three studies (27, 38, 47) did not account for the loss to follow-up, and their outcome data were incomplete. Eight studies (25–27, 34, 44, 45, 47, 48) did not provide relevant information about the study protocol, suggesting that there may have been selective reporting of findings.

### 3.3 Characteristics of study and participants

Twenty-eight studies were published between 2003 and 2024 and conducted in nine countries, namely, China (16, 17, 25, 26, 30, 34, 36, 43) (*n* = 8), the USA (27, 31, 32, 35, 37, 38, 42) (*n* = 7), the UK (44–46) (*n* = 3), Australia (33, 41, 47) (*n* = 3), Netherlands (48, 49) (*n* = 2), Korea (39, 40) (*n* = 2), Iran (50) (*n* = 1), India (28) (*n* = 1) and Turkey (29) (*n* = 1). A total of 3,163 stroke patients (Control group *n* = 1,559, Intervention group *n* = 1,604) were included in the review, and more than half of them (57.1%) were males. The sample size of these patients ranged from 10 (35) to 536 (48). In addition, 2,782 caregivers (control group *n* = 1,383, intervention group *n* = 1,399) were included, over 45.9% of whom were females. Most of studies had pairs of subjects, i.e., one patient to one caregiver, and only eight studies (27, 33, 41, 44–46, 48, 49) did not.

### 3.4 Characteristics of interventions

Details of interventions in included studies are summarized in Supplementary Table 2.

#### 3.4.1 Theoretical framework

In ten of the studies included (16, 17, 31, 33, 37, 41, 42, 47, 49, 50), various theoretical frameworks were adopted to guide the design of the interventions. Several theoretical frameworks focused on family, including Double ABC-X Model, Family Systems Theory and Family-Centered Empowerment Model. Additionally, the Stress and Coping Model, Stress Process Model, and Proactive Coping Theory concentrated on the individual’s stress-coping process. An exception was the Developmental-Contextual Model of Coping, which was a dyadic theory that considered the interaction between two individuals. Furthermore, the Health Belief Model, Collaborative Therapy Framework and Self-Efficacy Theory were also applied.

#### 3.4.2 Intervention elements

Among the studies included, interventions in twelve studies (16, 17, 25, 28, 35, 37, 43–45, 47, 48, 50) were provided by nurses, followed by therapists, psychologists, social workers and professionals, emphasizing clinical expertise. A variety of delivery modes of dyadic interventions were described. In eight studies (16, 17, 28, 36, 41, 43, 44, 48), interventions were delivered via face-to-face sessions plus telephone calls. In two studies (38, 45), interventions were administered via telephone calls. In eleven studies (25–27, 30, 34, 35, 39, 46, 47, 49, 50), interventions were given via face-to-face sessions. In other studies (*n* = 7), interventions were delivered via online platforms, such as email, video, or websites, complemented by in-person meetings or telephone conversations. All the interventions were delivered to single family dyads, but the intervention conducted by Tielemans (49) was administered to groups consisting of four to eight families. Most intervention programs were carried out in hospitals or at the patient’s residence, with one study’ intervention being conducted in nursing school (27).

The duration of dyadic interventions ranged from 4 days (50) to 9 months (46). Ten studies (26, 27, 31, 32, 34–36, 39, 40, 50) implemented four to sixteen sessions of either face-to-face or online education, with each session ranging from 20 min to 2 h in duration. Two studies (33, 49) offered initial common sessions followed by a final intensive session. Four studies (29, 38, 41, 45) provided telephone follow-ups ranging from 3 to 13 times, conducted either weekly or monthly. Two studies (37, 47) implemented interventions through multiple home visits, each spanning approximately 60 to 70 min. Six studies (16, 17, 28, 43, 44, 48) featured diverse formats and contents, with intervention durations ranging from 5 weeks to 6 months. These interventions combined in-person sessions, telephone consultations, and home visits. The details of interventions in the remaining four studies (25, 30, 42, 46) are not disclosed.

#### 3.4.3 Intervention contents

The interventions for stroke patients and their caregivers primarily encompassed the following aspects: offering tailored information support that catered to the needs of dyads (25, 48), including symptom management, strategies for preventing recurrence, medication-related information, physical rehabilitation, and other disease-related and self-care information (16, 28, 45); providing discharge education and referral services to bolster readiness for discharge and ease transitional recovery (43); training in relevant skills such as meditation (27) and narrative therapy (26) aimed at alleviating negative emotions and restoring a sense of purpose in life; communication skills training designed to foster mutual understanding and trust between the patient and caregiver (30, 31); and teaching coping skills equipped both parties to adapt and respond more effectively to the challenges posed by the disease (37, 44). Furthermore, ensuring adequate social support for the patient-caregiver dyad was essential for restoring social connections and facilitating social integration (29, 36, 49).

### 3.5 Control group

Majority of the included studies (*n* = 24) adopted routine care to be the control group, mainly encompassing health education, rehabilitation exercise, follow-up nursing, and management of complications. Two studies (27, 31) indicated that the control group received either expressive writing or information, support, and referral (ISR) intervention. Additionally, two studies (37, 49) provided the control group with stroke-related knowledge in the form of an information package.

### 3.6 Effectiveness of dyadic intervention on psycho-social outcomes for stroke patients and their caregivers

#### 3.6.1 Dyadic appraisal

Dyadic appraisal refers to patients’ and caregivers’ perceptions of the stressful situation, evaluation of internal and external resources, including caregiver-related burden, self-efficacy and family function. Among them, caregiver-related burden is the pressure experienced by caregivers when they perceive that patients’ needs exceed their personal care ability, which is a negative evaluation of the current stressful situation. Self-efficacy belongs to individual psychological resource, and family function belongs to external resources available to individuals. Therefore, these three indicators can be grouped into dyadic appraisal.

##### 3.6.1.1 Family function

**Figure 3:**
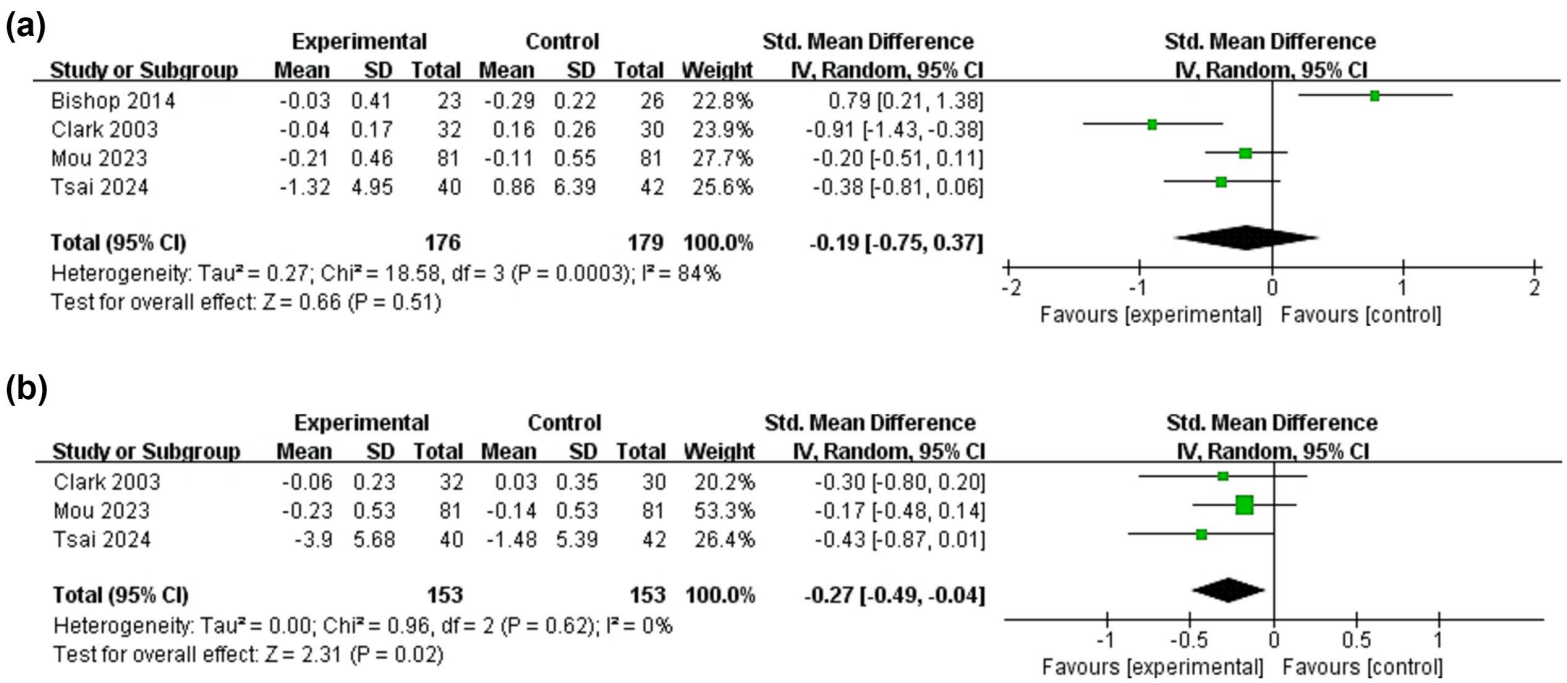
Forest plots: effectiveness of dyadic interventions on family function. **(a)** Patients; **(b)** Caregivers.

Four studies (16, 25, 38, 47) inquired into the impact of dyadic interventions on family function and could be included in the meta-analysis (Figure 3). The result of the random-effects model showed that dyadic intervention had no significant effect on family function of stroke patients (SMD = −0.19, 95%CI:−0.75 to 0.37, *p* = 0.51), with a relatively high heterogeneity (I^2^ = 84%, *p* = 0.0003). Subgroup analysis was not possible due to the small number of included literature. We then performed a sensitivity analysis and found that excluding Bishop’s with a high risk of bias (38), the pooled result of the remaining three showed the opposite conclusion (SMD = −0.45, 95%CI:−0.84 to −0.06, *p* < 0.05; I^2^ = 62%, *p* = 0.07). However, statistically significant improvements in caregivers’ family function occurred after the intervention (SMD = −0.27, 95%CI:−0.49 to −0.04, *p* < 0.05) without heterogeneity (I^2^ = 0%, *p* = 0.62).

##### 3.6.1.2 Caregiver-related burden

**Figure 4:**
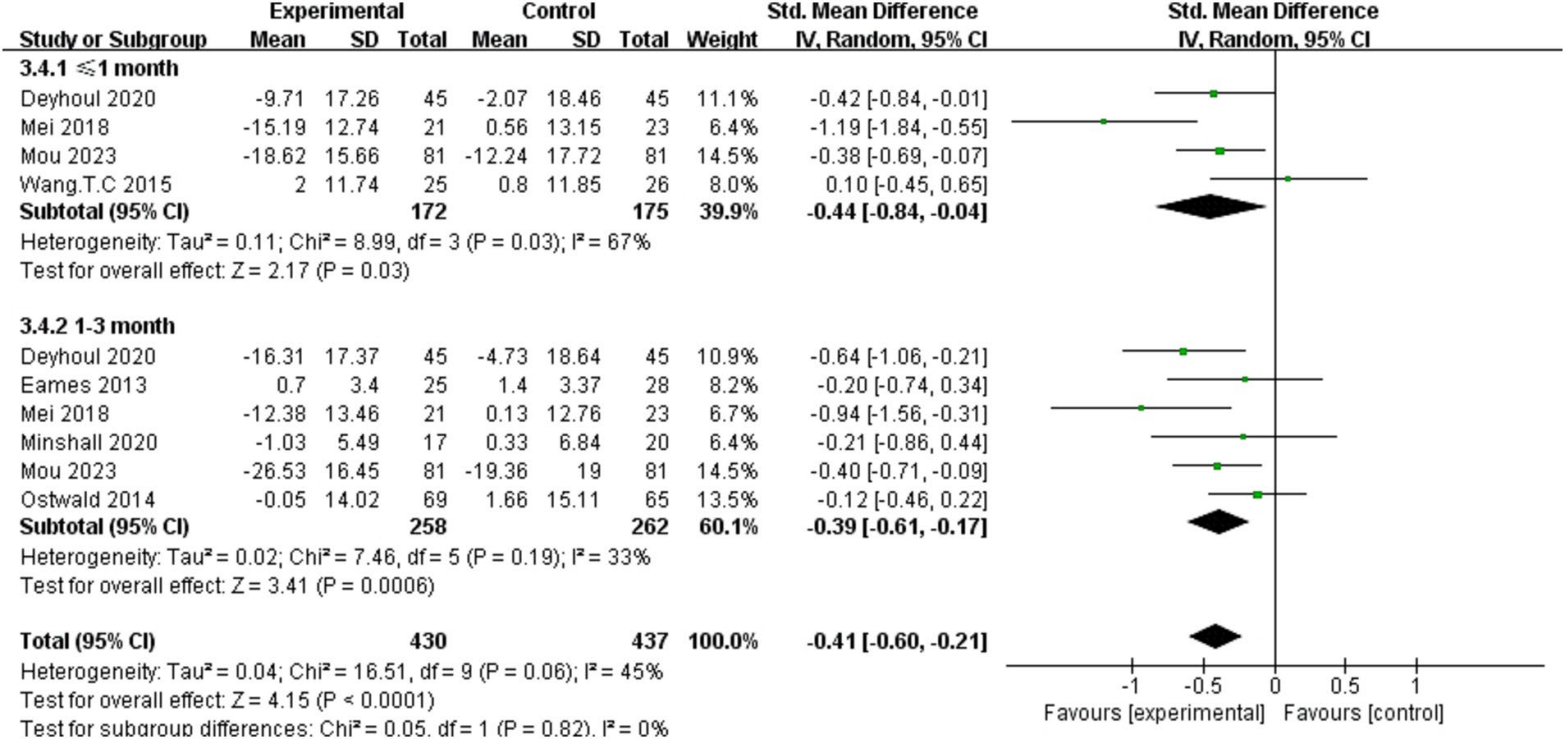
Forest plot: effectiveness of dyadic interventions on caregiver-related burden.

Fourteen studies (16, 17, 28, 30, 33, 34, 36, 37, 41, 45, 46, 48–50) inquired into the effect of dyadic interventions on caregivers’ burden, among seven studies (16, 33, 34, 36, 37, 41, 50) could be included in the meta-analysis (Figure 4). Subgroup analysis was conducted by time and the result of the random-effects model showed that dyadic interventions have a positive effect on caregivers’ burden 1 month post-intervention (SMD = −0.44, 95%CI:−0.84 to −0.04, *p* < 0.05) and the effect can be sustained up to 3 months after intervention (SMD = −0.39, 95%CI:−0.61 to −0.17, *p* < 0.05). There was no heterogeneity between subgroups (I^2^ = 0%, *p* = 0.82).

##### 3.6.1.3 Self-efficacy

**Figure 5:**
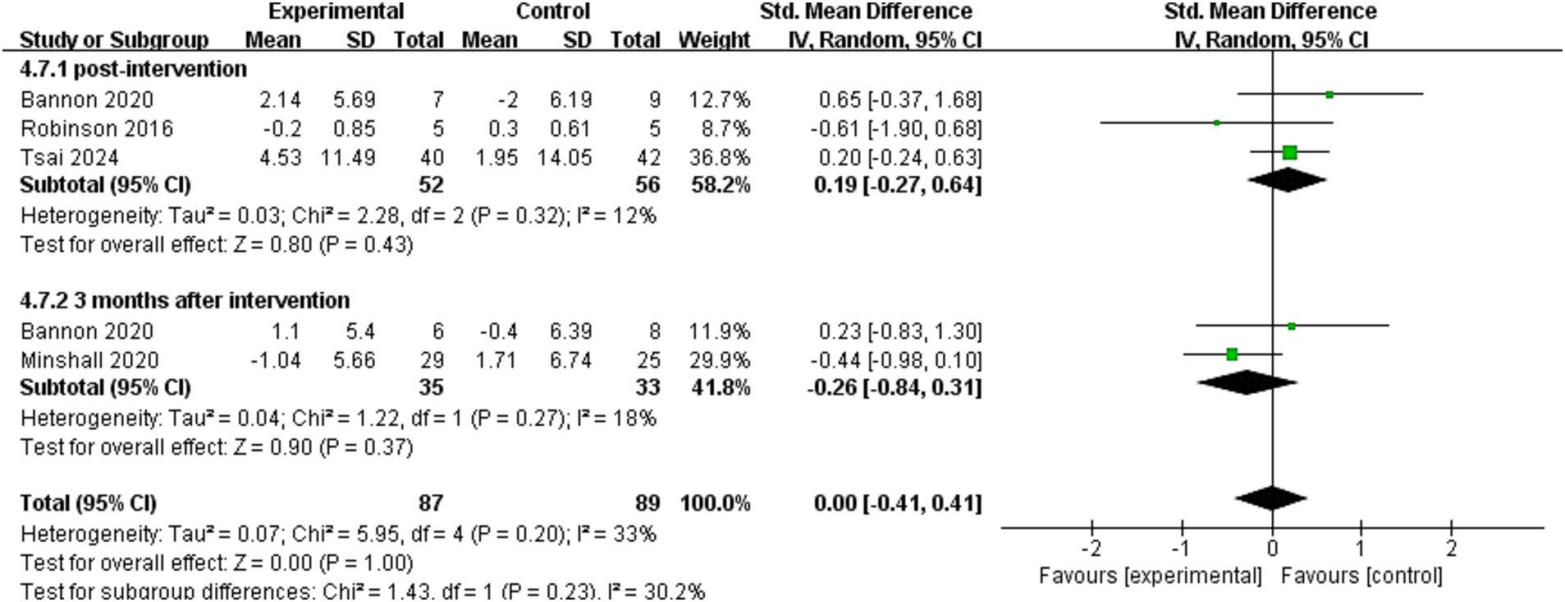
Forest plot: effectiveness of dyadic interventions on self-efficacy of patients.

Eight studies (17, 25, 29, 32, 33, 35, 41, 49) used patients’ self-efficacy as an outcome indicator, four (25, 32, 33, 35) of which could be included in meta-analysis (Figure 5). The results of pooled analyses were presented independently for immediate after intervention in three studies (25, 32, 35) and 3 months post-intervention in two studies (32, 33). The effect of dyadic intervention on patients’ self-efficacy was not significant (SMD = 0.00, 95%CI:−0.41 to 0.41, *p* = 1.00). Self-efficacy of caregivers was used as an outcome indicator in four studies (29, 32, 33, 49). Due to incomplete data, only synthesis without meta-analysis was performed. Three studies (29, 32, 33) suggested that self-efficacy of caregivers was not improved or maintained by the interventions, compared to the controls.

#### 3.6.2 Dyadic coping

**Figure 6:**
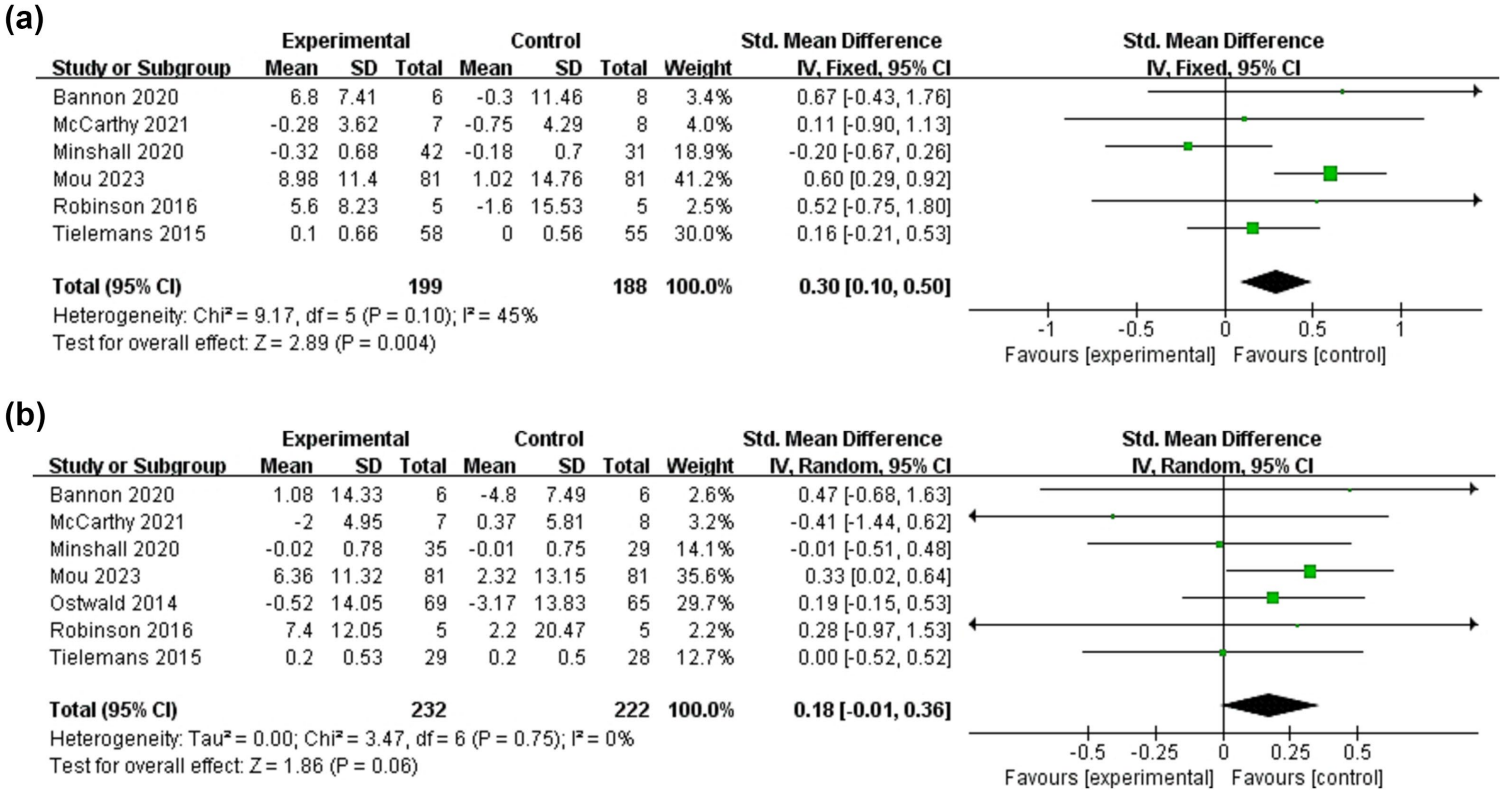
Forest plots: effectiveness of dyadic interventions on dyadic coping. **(a)** Patients; **(b)** Caregivers.

Dyadic coping refers to responses and strategies that are shared by both patients and their caregivers in the face of stress, which is mainly reflected in the outcome indicator of coping styles. Six studies (16, 31–33, 35, 49) investigated the effect of dyadic interventions on dyadic coping of patients and seven (16, 31–33, 35, 37, 49) examined that of caregivers (Figure 6). The result of the fixed-effects model showed that dyadic interventions had a beneficial effect on coping ability of patients (SMD = 0.30, 95%CI:0.10 to 0.50, *p* < 0.05) with a moderate heterogeneity (I^2^ = 45%, *p* = 0.10). However, there was no statistically significant improvement in caregivers’ coping after the intervention (SMD = 0.18, 95%CI:−0.01 to 0.36, *p* = 0.06).

#### 3.6.3 Dyadic adjustment

Dyadic adjustment is ultimate goal of the developmental contextual coping model, which is the adjustment and adaptation of the physical, psychological and social aspects of both parties after coping with stress and consists mainly of functional independence, depression, quality of life and relationship quality. Among them, functional independence is the physiological goal, depression and relationship quality are the indicators of psychological adjustment, and the improvement of quality of life is the comprehensive goal achieved by the implementation of intervention.

##### 3.6.3.1 Functional independence

**Figure 7:**
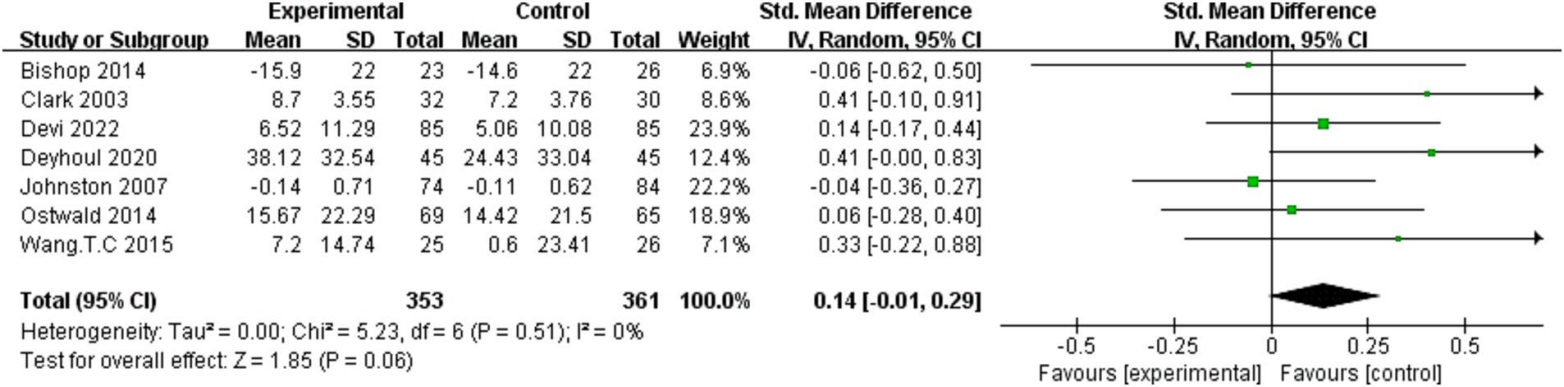
Forest plot: effectiveness of dyadic interventions on functional independence of patients.

Eleven studies (16, 28, 30, 36–38, 44, 45, 47, 48, 50) measured patients’ functional independence as an important outcome of which seven studies (28, 36–38, 44, 47, 50) could be pooled for meta-analysis (Figure 7). The result of the random-effects model showed that the effect of dyadic intervention on patients’ functional improvement was not statistically significant (SMD = 0.14, 95%CI:−0.01 to 0.29, *p* = 0.06) with no heterogeneity (I^2^ = 0%, *p* = 0.51). Synthesis without meta-analysis showed that dyadic intervention could improve functional independence of patients (30).

##### 3.6.3.2 Quality of life

**Figure 8:**
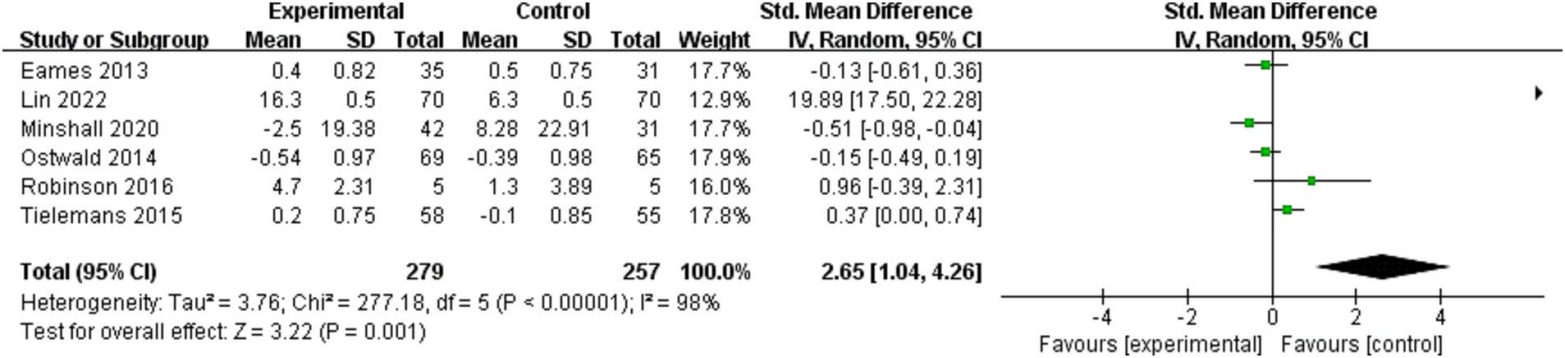
Forest plot: effectiveness of dyadic interventions on quality of life of patients.

Seven studies (25, 29, 30, 33, 37, 44, 47) investigated the effect of dyadic interventions on quality of life in caregivers. Owing to incomplete data, only synthesis without meta-analysis was performed. Overall, quality of life of caregivers was not significantly improved (25, 33, 44, 47). Twelve studies (17, 25, 28–30, 33, 35, 37, 41, 47–49) investigated that of patients, of which six (17, 33, 35, 37, 41, 49) could be included in the meta-analysis (Figure 8). The result of the random-effects model showed that dyadic intervention had a statistically significant improvement in quality of life of patient (SMD = 2.65, 95%CI:1.04 to 4.26, *p* < 0.05) but high heterogeneity existed (I^2^ = 98%, *p* < 0.00001). Subgroup analyses could not be performed due to the small number of included studies and incomplete data. To seek further sources of heterogeneity, we performed a sensitivity analysis and found that excluding Lin’s study (17) reduced heterogeneity dramatically (I^2^ from 98 to 64%), yet yielded negative results (SMD = −0.03, 95%CI:−0.39 to 0.34, *p* = 0.89).

##### 3.6.3.3 Depression

**Figure 9:**
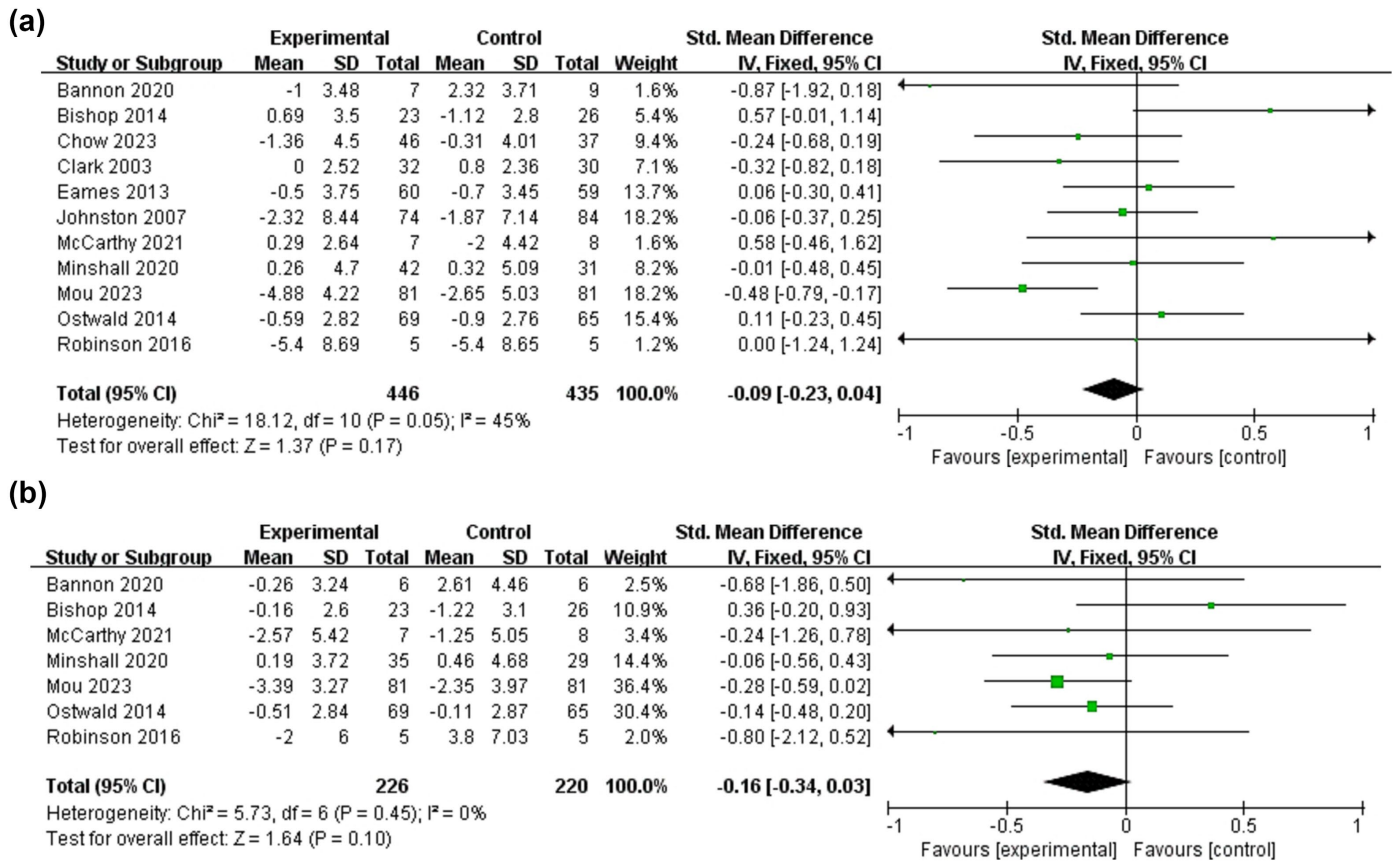
Forest plots: effectiveness of dyadic interventions on depression. **(a)** Patients; **(b)** Caregivers.

Sixteen studies (16, 26, 27, 30–33, 35, 37, 38, 41, 42, 44, 45, 47, 48) investigated the effect of dyadic interventions on depression in patients, of which eleven studies (16, 26, 31–33, 35, 37, 38, 41, 44, 47) could be included in the meta-analysis (Figure 9). The result of the fixed-effects model showed that dyadic interventions has no positive effect on the patients’ depression (SMD = −0.09, 95%CI:−0.23 to 0.04, *p* = 0.17) and a moderate heterogeneity existed (I^2^ = 45%, *p* = 0.05). Depression of caregivers was chosen as an outcome indicator in eleven studies (16, 26, 27, 30–33, 35, 37, 38, 42), of which seven studies (16, 31–33, 35, 37, 38) could be included in the meta-analysis. The result showed that caregivers’ depression was also not improved by dyadic intervention (SMD = −0.16, 95%CI:−0.34 to 0.03, *p* = 0.10) without heterogeneity (I^2^ = 0%, *p* = 0.45).

##### 3.6.3.4 Relationship quality

**Figure 10:**
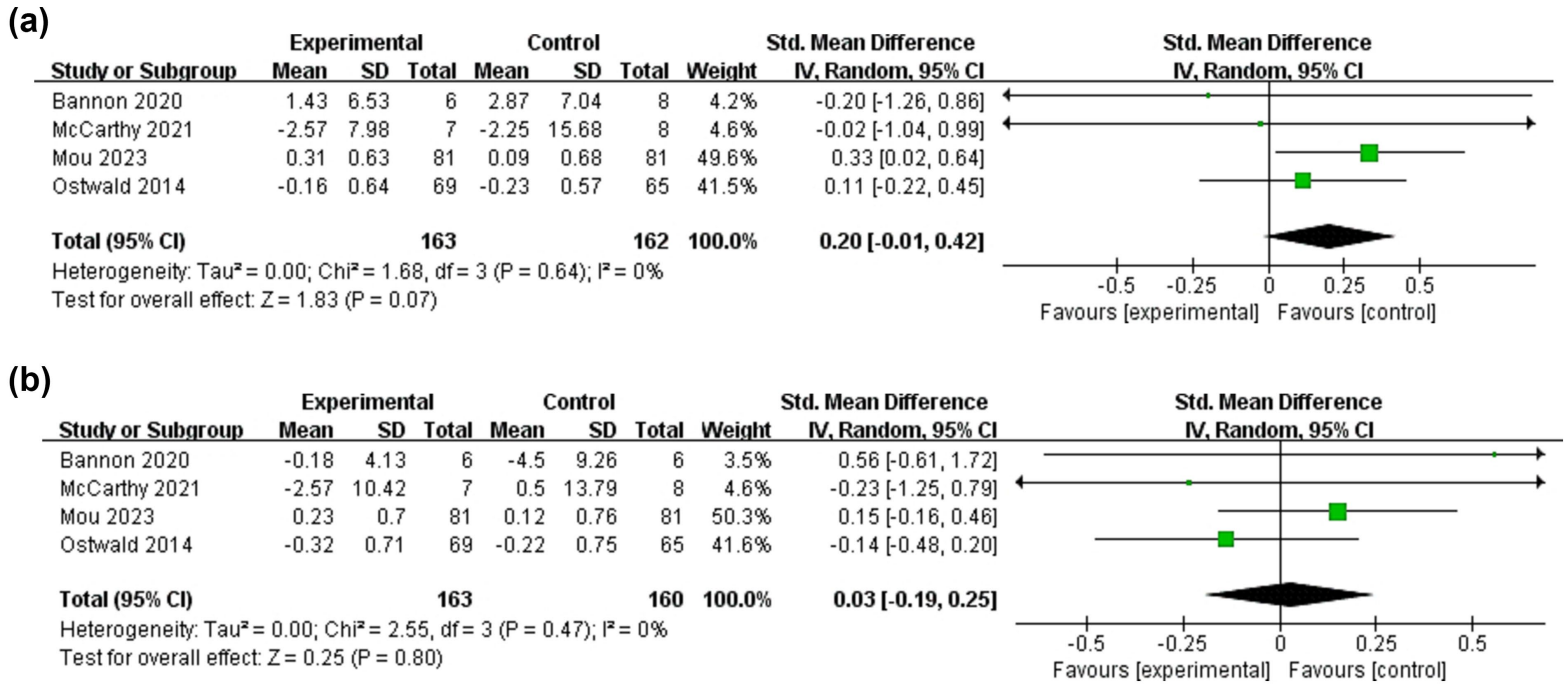
Forest plots: effectiveness of dyadic interventions on relationship quality. **(a)** Patients; **(b)** Caregivers.

Relationship quality of stroke patients and caregivers was used as an outcome indicator in four studies (16, 31, 32, 37) (Figure 10). The results of meta-analysis showed that there was no significant difference in the relationship quality of patients between the intervention and control groups (SMD = 0.20, 95%CI:−0.01 to 0.42, *p* = 0.07) and no heterogeneity (I^2^ = 0%, *p* = 0.64). The same was true for caregivers (SMD = 0.03, 95%CI:−0.19 to 0.25, *p* = 0.80; I^2^ = 0%, *p* = 0.47).

## 4 Discussion

Dyadic interventions view stroke patients and their caregivers as a whole, taking into account their interdependence and interactions, and improve the effectiveness of intervention program and patient compliance by enhancing relationship quality and interaction between dyads. On the form of intervention, a combination of delivery modes is more effective than a single form (51). For example, the delivery mode combining of face-to-face, telephone and network breaks the time and geographical restrictions, provides continuous support outside the hospital, and makes the subjects feel more flexible and autonomous while ensuring the intervention effect (52). Also, although it has been reported that group intervention can be more effective by giving participants the opportunity to meet with other dyads and to share their experiences of disease with people in similar situations (22), only one study (49) involved group approach, which included peer support. At present, there is no unified standard for intervention intensity, which can be flexibly adjusted according to subjects’ preferences and disease conditions to maximize interventions’ benefits. Additionally, most studies did not achieve personalized intervention. Considering differences in personality and disease characteristics of stroke patients and their caregivers, they face different difficulties and needs in coping with the disease. In the future, qualitative research methods can be used to interview the dyads, explore needs and preferences of the dyads, and develop practical and demand-oriented interventions.

The results of meta-analysis showed differences in the impact of dyadic interventions on patients and caregivers. Self-efficacy and family function are important psychological resources for patients and their caregivers, influencing dyads’ coping ability and confidence in recovery. The improvement of psychological resources can promote active cooperation with the treatment and maintain optimism, so as to better cope with the disease. However, the effect of interventions on dyads’ self-efficacy was not significant. Possible explanations are that the physical and psychological sequelae of stroke cause patients to lose confidence in recovery, as well as the fear of recurrence of the disease also limits the improvement of self-efficacy. The patient’s poor state triggers caregivers’ concerns about their own caregiving ability, which to some extent also limits the improvement of caregiver self-efficacy (29). The results coincide with the conclusions of a meta-analysis (53). In addition, dyadic intervention positively impacted caregivers’ family functioning, because interventions involve the identification and resolution of family problems, facilitating communication between patients and caregivers, while mobilizing other family members to provide material help and emotional support for the dyads. There was no statistically significant improvement in family function perceived by patients. However, the pooled result is positive after excluding Bishop’s study (38). It may be that control group in the study used standard medical follow-up, distinguishing it from the routine care of other studies, led to the non-significant result.

The pooled results of seven studies showed that dyadic interventions were not statistically effective in improving patients’ functional independence, which is inconsistent with the finding of some single studies (45, 50). The reason may be that the intervention is particularly effective in the optimal recovery period of 6 months after a stroke (54), and five (28, 37, 38, 44, 47) of the seven included studies evaluated functional independence after 6 months post-intervention, missing the optimal rehabilitation period and leading to a decline in the patient’s physical functioning. Future studies should measure the indicator during the optimal recovery period to validate the effectiveness of the intervention. Caregivers often experience greater burden due to patients’ dependence, heavy caregiving tasks and inadequate support systems. Caregiver burden decreases as patient’s function restores. So, this review concluded that dyadic interventions significantly lightened caregiver-related burden. Simultaneously, as a key factor for stroke families, functional independence can also influence other psycho-social outcomes, such as quality of life of dyads (55). We found that dyadic interventions can significantly improve quality of life for patients, but not for caregivers, the reason for which may be caregivers instinctively put needs and health of patients in the first place, thus ignoring themselves. However, when removing Lin’s study (17), the effect of interventions on patients’ quality of life was negative. The reason is that Lin’s study used the total score of the scale, while other studies used the average score of items. It is suggested that future intervention design should balance both the interests of dyads and the independent needs of each individual to scale up the benefits of intervention.

The review also found that dyadic intervention had no positive effects on the depression and relationship quality of patients and their caregivers. Although the incidence of depression in stroke patients and caregivers is relatively high (7, 10), the lower levels of depression at baseline in dyads in most of the included studies made it difficult to show a positive effect of the intervention. As for relationship quality, theoretically dyadic interventions emphasize the importance of communication between dyads, but show non-significant result. It may be that both the patient and the caregiver are burdened with different pressures and choose to empathize with each other in silence in order not to further add to each other’s burdens. Future interventions should therefore focus on more diverse sample with different levels of depression and value emotional expression between dyads.

Currently, outcome indicators are limited to the feasibility and benefits of interventions, with less consideration of safety and health economic indicators. Only two studies (17, 48) considered the occurrence of adverse events and unplanned readmissions. The results showed that interventions prompted a decreasing trend in the numbers of adverse events and lower rate of unplanned hospital readmissions. Due to small number of studies, the results are not convincing. In the future, we can further improve the effectiveness evaluation system of dyadic intervention, so that it can better integrate with the clinical environment and patient demands.

### 4.1 Limitation

There are several limitations in this study. Firstly, the review only included literature in English, which may introduce selection bias, particularly for region-specific studies published in local languages. Future reviews could benefit from multilingual search strategies. Secondly, most of studies included did not blind the subjects or intervenors, leading to a certain degree of risk of bias, which is uncontrollable due to the openness of clinical trials. Future studies could attempt to select patients and caregivers from each of the two wards for the intervention to ensure that blinding is implemented. Finally, some of included studies had a higher risk of bias, affecting the accuracy of the evidence. Future research should suggest more stringent inclusion and exclusion criteria to screen high-quality literature.

## 5 Conclusion

On the whole, these findings highlight the positive impact of dyadic interventions on stroke patients and their caregivers. However, effectiveness of interventions targeting some indicators was not significant and need to be further verified. In the future, RCTs with high-quality study designs are recommended to validate the effectiveness of dyadic intervention programs for stroke patients and their caregivers. Meanwhile, we are committed to finding new interventionable elements and developing demand-oriented personalized intervention measures to provide guarantee for the implementation of clinical dyadic intervention.

## Data Availability

The original contributions presented in the study are included in the article/Supplementary material, further inquiries can be directed to the corresponding author.
