# Supplementary material for: The impact of dyadic interventions on psycho-social outcomes for stroke patients and their caregivers: a systematic review and meta-analysis
Source: Front Public Health. 2025 May 9;13:1583621. doi: 10.3389/fpubh.2025.1583621 (PMC12098548; doi:10.3389/fpubh.2025.1583621)
Supplement: Supplementary file 1 [file Data_Sheet_1.docx]

Supplementary Material

# Supplementary Figures and Tables

## Supplementary Table 1 Search strategy

**PubMed (n=1821)**

| #1: cerebral infarction [MeSH Terms] OR cerebral hemorrhage [MeSH Terms] OR cerebrovascular disorders [MeSH Terms] OR stroke [MeSH Terms] OR hemorrhagic stroke [MeSH Terms] OR embolic stroke [MeSH Terms] OR thrombotic stroke [MeSH Terms] OR ischemic stroke [MeSH Terms] OR brain infarction [MeSH Terms] |
| --- |
| #2: cerebrovascular disease [Title/Abstract] OR cerebral thrombosis [Title/Abstract] OR apoplexy [Title/Abstract] |
| #3: #1 OR #2 |
| #4: caregivers [MeSH Terms] OR spouses [MeSH Terms] |
| #5: partner* [Title/Abstract] OR couple* [Title/Abstract] OR dyad* [Title/Abstract] OR dyadic [Title/Abstract] |
| #6: #4 OR #5 |
| #7: cognitive behavioral therapy [MeSH Terms] OR behavior therapy [MeSH Terms] OR psychosocial intervention [MeSH Terms] OR health education [MeSH Terms] OR rehabilitation [MeSH Terms] OR knowledge [MeSH Terms] |
| #8: intervention [Title/Abstract] OR dyadic coping [Title/Abstract] OR dyadic management [Title/Abstract] OR support* [Title/Abstract] OR psychoeducat* [Title/Abstract] OR cognitive therapy [Title/Abstract] |
| #9: #7 OR #8 |
| #10: #3 AND #6 AND #9 |

**Web of Science (n=2170)**

| #1: stroke (TS) OR cerebral infarction (TS) OR cerebral hemorrhage(TS) OR cerebrovascular disease (TS) OR apoplexy (TS) |
| --- |
| #2: caregivers (TS) OR partner* (TS) OR spouse* (TS) OR couple* (TS) OR dyad* (TS) OR dyadic (TS) |
| #3: dyadic intervention (TS) OR dyadic coping (TS) OR dyadic management (TS) OR cognitive behavioral therapy (TS) OR psychosocial intervention (TS) OR health education (TS) OR rehabilitation (TS) |
| #4: #1 AND #2 AND #3 |

**CINAHL** **(n=479)**

| #1: stroke (MH) OR ischemic stroke (MH) OR hemorrhagic stroke (MH) OR cerebral infarction (MH) OR cerebral hemorrhage (MH) OR cerebrovascular disorders (MH) |
| --- |
| #2: cerebrovascular disease (TI) OR cerebrovascular disease (AB) OR apoplexy (TI) OR apoplexy (AB) |
| #3: #1 OR #2 |
| #4: caregivers (MH) OR spouses (MH) |
| #5: partner (TI) OR partner (AB) OR couples (TI) OR couples (AB) OR dyad (TI) OR dyad (AB) OR dyadic (TI) OR dyadic (AB) |
| #6: #4 OR #5 |
| #7: psychosocial interventions (MH) OR health education (MH) OR rehabilitation (MH) |
| #8: dyadic intervention (TI) OR dyadic intervention (AB) OR dyadic coping (TI) OR dyadic coping (AB) OR dyadic management (TI) OR dyadic management (AB) OR cognitive behavioral therapy (TI) OR cognitive behavioral therapy (AB) OR intervention (TI) OR intervention (AB) |
| #9: #7 OR #8 |
| #10: #3 AND#6 AND #9 |

**Embase (n=1869)**

| #1: cerebrovascular accident (MH) OR brain infarction (MH) OR brain hemorrhage (MH) |
| --- |
| #2: stroke (ti, ab, kw) OR cerebral infarction (ti, ab, kw) OR cerebral hemorrhage (ti, ab, kw) OR cerebrovascular disease (ti, ab, kw) OR apoplexy (ti, ab, kw) |
| #3: #1 OR #2 |
| #4: caregiver (MH) OR spouse (MH) |
| #5: dyad* (ti, ab, kw) OR dyadic (ti, ab, kw) |
| #6: #4 OR #5 |
| #7: dyadic coping (MH) OR cognitive behavioral therapy (MH) OR health education (MH) OR rehabilitation (MH) OR psychosocial intervention (MH) |
| #8: dyadic intervention (ti, ab, kw) OR dyadic management (ti, ab, kw) |
| #9: #7 OR #8 |
| #10: #3 AND #6 AND #9 |

**Cochrane Library (n=262)**

| #1: stroke (MeSH) OR cerebral infarction (MeSH) OR cerebral hemorrhage (MeSH) OR cerebrovascular disorders (MeSH) |
| --- |
| #2: apoplexy (ti, ab, kw) OR cerebrovascular disease (ti, ab, kw) |
| #3: #1 OR #2 |
| #4: caregivers (MeSH) OR spouses (MeSH) |
| #5: partner (ti, ab, kw) OR couple (ti, ab, kw) OR dyad (ti, ab, kw) OR dyadic (ti, ab, kw) |
| #6: #4 OR #5 |
| #7: cognitive behavioral therapy (MeSH) OR psychosocial intervention (MeSH) OR rehabilitation (MeSH) |
| #8: dyadic intervention (ti, ab, kw) OR dyadic coping (ti, ab, kw) OR dyadic management (ti, ab, kw) OR health education (ti, ab, kw) |
| #9: #7 OR #8 |
| #10: #3 AND #6 AND #9 |
